# TT-TG distance decreases after open wedge distal femoral varization osteotomy in patients with genu valgum & patellar instability. A pilot 3D computed tomography simulation study

**DOI:** 10.1186/s12891-023-06832-w

**Published:** 2023-09-25

**Authors:** Volkan Gür, Furkan Yapici, İzzet Özay Subaşi, Mehmet Burak Gökgöz, Oğuzhan Tanoğlu, Nizamettin Koçkara, Nevzat Reha Tandoğan

**Affiliations:** 1 Department of Orthopedics, Erzincan Faculty of Medicine, Erzincan, Turkey; 2 Department of Orthopedics, Buca Seyfi Demirsoy Training and Research Hospital, İzmir, Turkey; 3 Department of Orthopedics, Haliç University Faculty of Medicine, İstanbul, Turkey

**Keywords:** Genu Valgum, Patellar instability, TT-TG distance, Osteotomy, 3D Simulation

## Abstract

**Background:**

Genu valgum is considered to be a risk factor for patellar instability. Correction of valgus alone or combined with a tibial tuberosity medialization has been described. However, changes occurring in the tibial tuberosity-trochlear groove (TT-TG) distance after a lateral distal femoral opening wedge osteotomy (LDF-OWO) are not known.

**Purpose:**

This study aimed to define changes in TT-TG distance with increasing amounts of valgus correction after LDF-OWO.

**Methods:**

Three-Dimensional Computed Tomography (3D-CT) scans of six patients (two females and four males) aged between 19 and 35 years with genu valgum and patellar instability were analyzed. 3D models of femoral, tibial, and patellar bones were created with the advanced segment option of Mimics 21^®^ software. An oblique lateral opening wedge osteotomy of the distal femur was simulated in 2-degree increments up to 12 degrees of varus opening. Change in TT-TG distance was measured in mm on 3D models of the knee.

**Results:**

Compared to baseline without osteotomy, the TT-TG distance decreased significantly (p < 0.05) for all corrections from 2 to 12 degrees in 2° steps. The TT-TG distance decreased by an average of 1.7 mm for every 2 degrees of varus opening.

**Conclusion:**

Lateral opening wedge distal femoral osteotomy causes a decrease in TT-TG distance. The surgeon should be aware of the magnitude of this change (1.7 mm decrease for every 2° increment of valgus opening).

**Supplementary Information:**

The online version contains supplementary material available at 10.1186/s12891-023-06832-w.

## Introduction

Genu valgum is considered a risk factor for recurrent patellar instability in adolescents and adults [1]. Although clear cut-off does not exist, correcting excessive knee valgus may be indicated in patients with patellar instability. The deformity is frequently in the distal femur, and several methods, including guided growth techniques, open and closed wedge distal femoral osteotomies, have been described to correct valgus. These can be performed in isolation or combined with an MPFL reconstruction and/or tibial tuberosity transfers [2].

The Tibial Tuberosity-Trochlear Groove (TT-TG) Distance is widely used to objectively measure the increased lateral quadriceps vector in patients with patellar instability. First described by Dejour, the TT-TG distance was found to be 19.8 mm in patients with patellar instability compared to 12.7 mm in normal knees. A tibial tuberosity medialization osteotomy is indicated for patients with > 20 mm TT-TG, while 15–20 mm range is considered to be the “grey zone” [3]. The effect of distal femoral osteotomy on TT-TG distance has not been adequately addressed. Most studies have been performed on medial closing wedge osteotomies, with some studies finding change while others finding no effect of distal femoral osteotomy on TT-TG distance [4–6]. This is an important knowledge gap in the treatment of patellar instability treatment, as additional tibial tuberosity osteotomy may no longer be necessary after leg axis correction.

This study aimed to analyze changes in the TT-TG distance after a Lateral Distal Femoral Opening Wedge Osteotomy (LDF-OWO) in a computer simulation study using 3 Dimentional Computed Tomography (3D-CT) scans of patients with patellar instability and genu valgum. We hypothesized that the TT-TG distance would decrease after LDF-OWO.

## Methods

Supine 3D CT scans of six adults (two females and four males) with patellar instability and genu valgum between 19 and 35 years of age were analyzed. We excluded patients with prior deformity correction or fracture fixation, incomplete imaging of the knee, and knee arthritis. A whole leg standing orthoroentgenogram was performed to evaluate the mechanical axis of the lower leg and the degree of genu valgum. CT scans were performed on a Siemens SOMATOM Emotion (Siemens, Erlangen, Germany) (110 kV-90 mAs, slice thickness 1.2 mm.). Anonymization of the patients’ demographic data was performed with the software Mimics 21 (Materialise, Leuven, Belgium).

3D models of femoral, tibial, and patellar bones were created with the advanced segment option of Mimics 21. Thresholding limits were determined as 200–2150 Hounsfield Unit to isolate the bone tissue from the medullary canal and soft tissues as previously described [7]. An incomplete oblique lateral opening wedge distal femoral osteotomy, starting 2 cm superior to the lateral epicondyle and aiming distally to the adductor tubercle, was created on 3D femoral models as described by Matthew et al. [8]. This osteotomy configuration was chosen as it was the most common method of open wedge distal femoral osteotomy. 3D models were transferred to Materialise 3-Matic 13 (Materialise, Leuven, Belgium). The distal femoral fragment and the tibia were merged into a single bone to ensure they moved simultaneously during the varus opening. The interactive rotation/translation option of the 3-Matic software was used to avoid unintended rotation or translation during varus displacement of the distal osteotomy fragment.

**Fig. 1:**
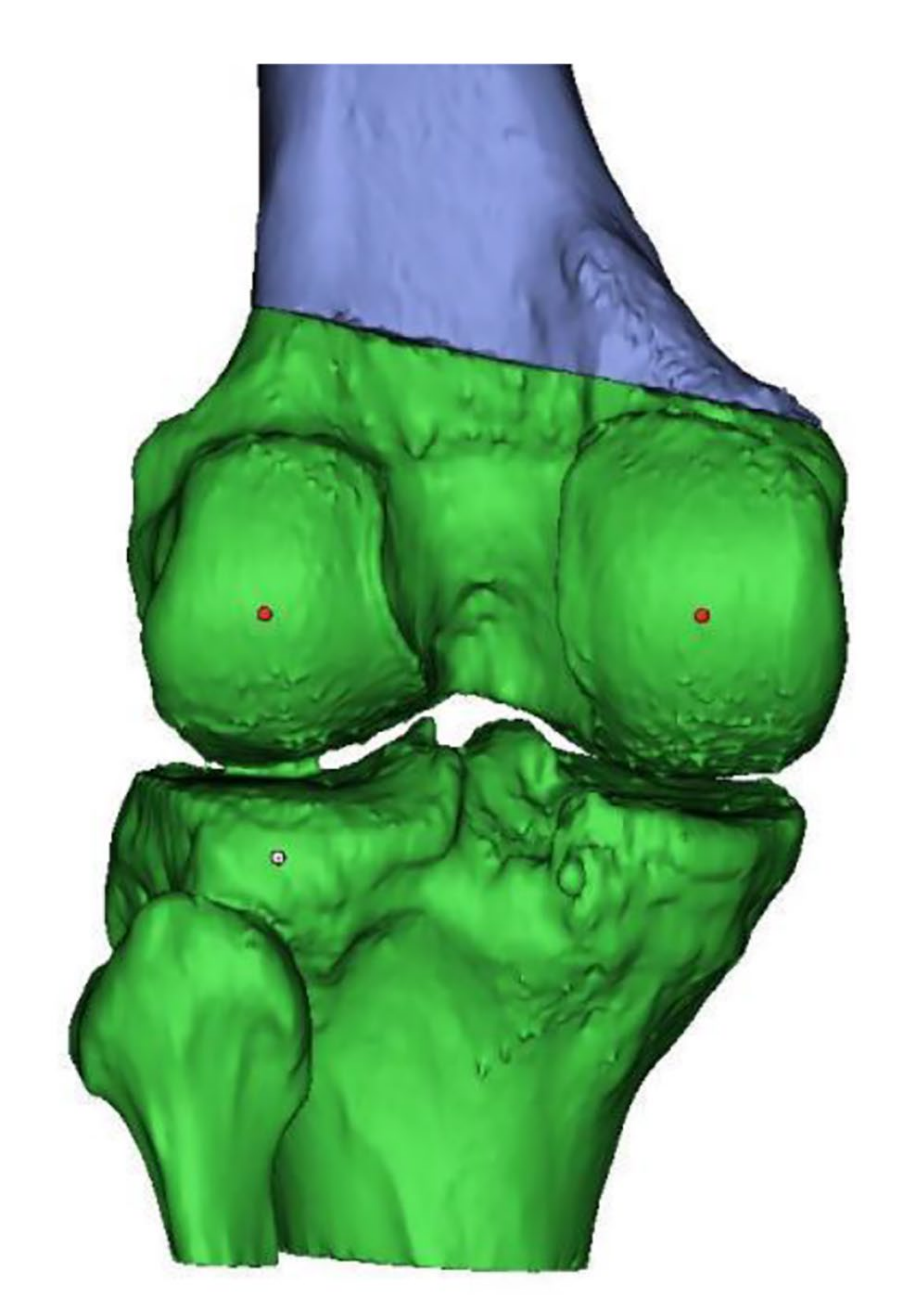
Determination of the most posterior points of medial and lateral femoral condyles

**Fig. 2:**
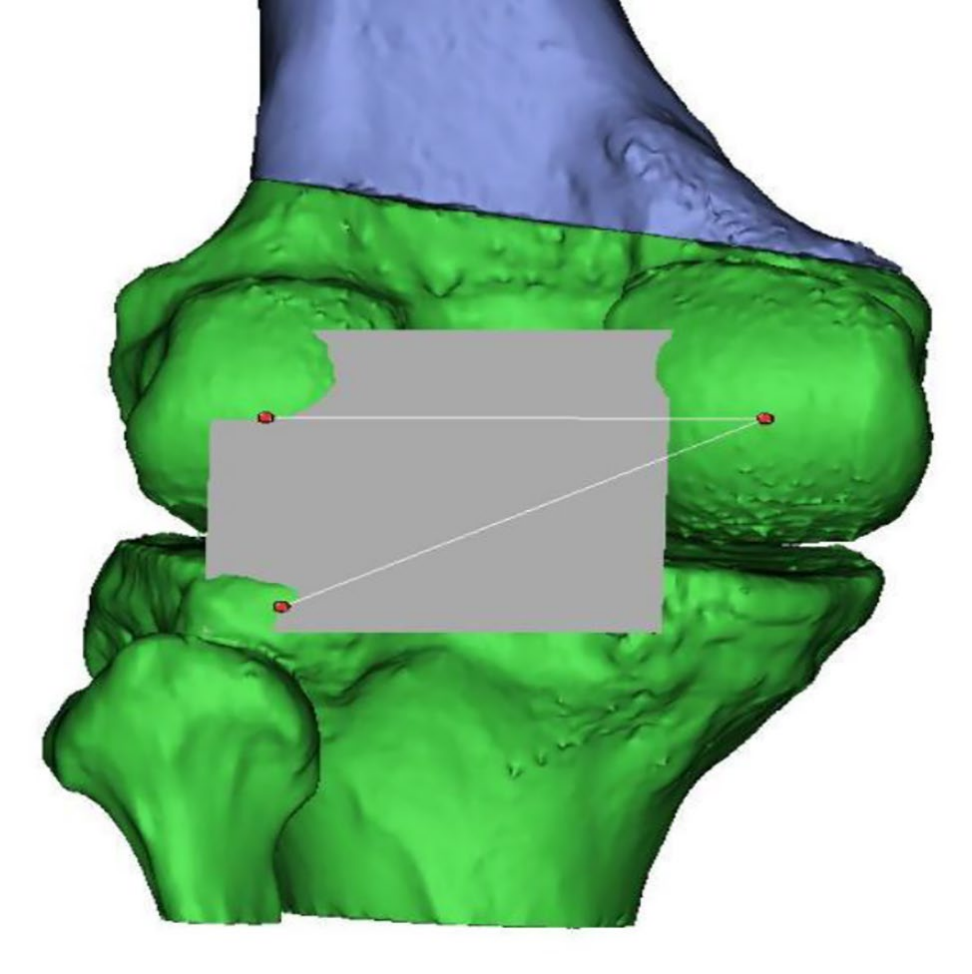
Creating an anatomical plane according to the most posterior points of medial femoral condyle, lateral femoral condyle and lateral tibial plateau

**Fig. 3:**
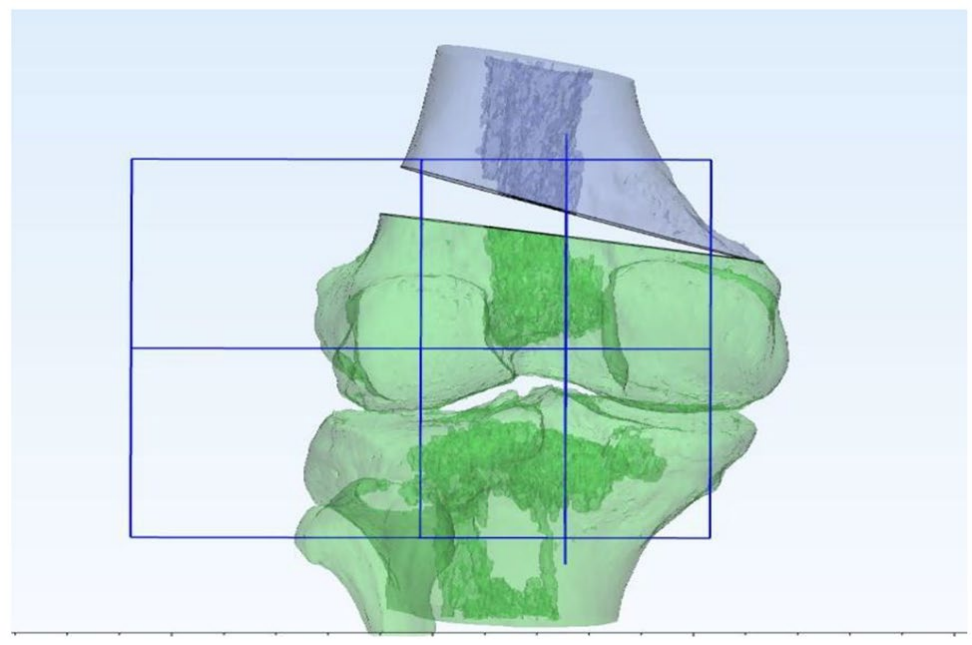
Posterior anatomical plane and the perpendicular plane of posterior anatomical plane (Anteroposterior view)

**Fig. 4:**
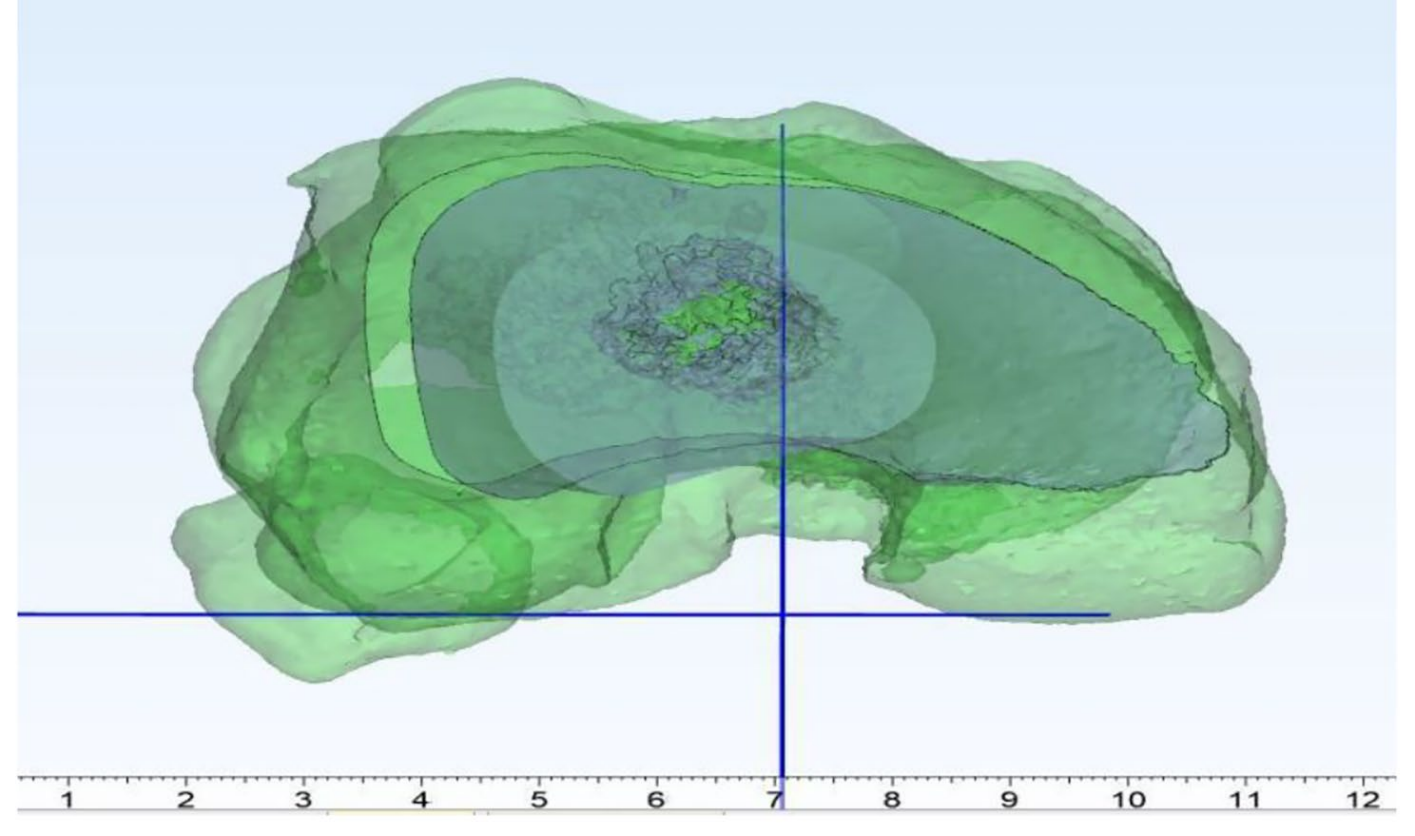
Posterior anatomical plane and the perpendicular plane of posterior anatomical plane (view from top)

The trochlea’s deepest point and the tibial tuberosity’s midpoint were marked using create point option of 3-Matic. The most posterior points of the lateral tibial condyle, medial, and lateral femoral condyles were marked on 3D models. The posterior condylar line in the XY axis, referencing the most posterior points of medial and lateral femoral condyles, was created [9]. A plane according to these three points was created using create datum plane option. A midplane that is perpendicular to the predefined posterior plane was created using the same option of the program. These two perpendicular planes were used to avoid any rotation or translation during opening wedge osteotomy. (Figures 1, 2, 3 and 4) The native TT-TG distance was measured using the automated ruler of 3-Matic.

**Fig. 5:**
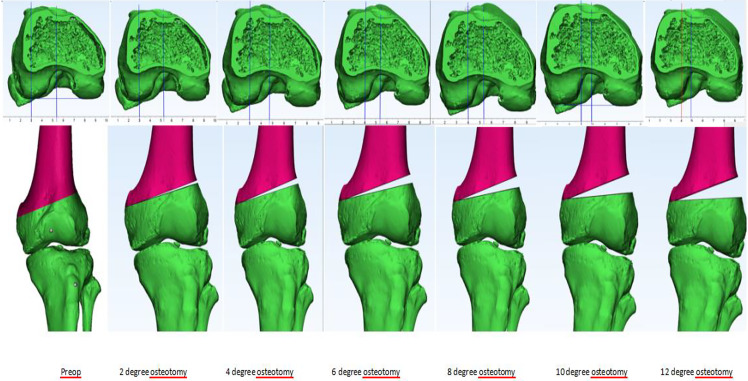
Demonstration of TT-TG distance measurement after LDF-OWO with 2° increments from baseline to 12°. A decrease in TT-TG distance is observed as the amount of varization increases

Using the interactive rotate option of 3-Matic, the distal part of the femur below the osteotomy line was rotated 2 degrees in the coronal plane to perform a varus opening. The pivot point of opening was selected as 1 cm from the medial cortex at the apex of the osteotomy. The posterior condylar line and the posterior plane with a perpendicular midplane were created again using the same method. Midplanes were created and translated as passing through the previously defined points. The distances were measured using the automated ruler of 3-Matic. This procedure was repeated for all TT-TG measurements performed in 2-degree increments from 2 to 12 degrees varus opening (Fig. 5).

A single observer, experienced in the use of the 3D simulation software performed all measurements.

### Statistical analysis

Mean, standard deviation, median, minimum, maximum value frequency, and percentage were used for descriptive statistics. The distribution of variables was checked with the Shapiro-Wilk normality test. Wilcoxon test was used for the repeated measurement analysis. All statistical analyzes were performed using SPSS 28.0 (SPSS Inc, Chicago, IL, USA). A p-value < 0.05 was considered statistically significant.

## Sample size calculation

We determined the threshold probability for rejecting the null hypothesis as α (two-tailed) = 0.05 (Type I error rate), probability of failing to reject the null hypothesis under the alternative hypothesis as a β = 0.20 (Type II error rate), and after determining expected correlation coefficient r ≥ 0.9, we found the minimum number of cases to be 6.

### Ethical aspects

This observational study was carried out with the approval of the Institutional Review Board of Ankara City Hospital No. 1 clinical research ethics committee (Date: 11.11.2020, Number: E1-20-1309). All experimental protocols were approved by this ethics committee. All patients gave informed consent to their anonymized data being used in the study. All methods were carried out in accordance with relevant guidelines and regulations.

## Results

The mean pre-osteotomy HKA of the patients was 3,2° (range, 1.5-6°). Mean LDFA and MPTA were 82,2° and 87,7° respectively. All patients reported at least one episode of patellar instability.

The mean TT-TG distance was 21 mm. All patients had a TT-TG distance above 15 mm, and three patients had a TT-TG > 20 mm.

Following simulated LDF-OWO, the TT-TG distance demonstrated a significant decrease for every 2° increase in distal femoral varus opening from 2° to 12° compared to baseline (p < 0.05). The differences in increments of 2 degrees were statistically significant for all values except for the differences between 6 and 8 degrees and 10 and 12 degrees (Table 1).

**Table 1:** Comparison of TT-TG distance between every 2° increase in distal femoral varus opening from baseline to 12°

		TT-TG Distance (mm)							p*		p‡	
		Min-Max			Median	Mean ± sd						
Distal Femoral Osteotomy Angle	Preop	18.0	-	24.0	21.0	21.0	±	2.1				
	2º	17.0	-	22.0	19.0	19.2	±	2.0	***0.026***	^w^		
	4º	15.0	-	19.0	16.0	16.3	±	1.5	***0.026***	^w^	***0.027***	^w^
	6º	14.0	-	17.0	15.0	15.2	±	1.2	***0.027***	^w^	0.066	^w^
	8º	12.0	-	17.0	13.0	13.7	±	1.8	***0.027***	^w^	***0.042***	^w^
	10º	9.0	-	13.0	12.0	11.5	±	1.4	***0.027***	^w^	0.102	^w^
	12º	9.0	-	12.0	11.5	10.8	±	1.5	***0.027***	^w^	***0.041***	^w^

***TT-TG***: Tibial Tuberosity – Trochlear Groove

p*: Difference with preop / p ‡ Difference with previous measurement

^***w***^ : Wilcoxon test

**Fig. 6:**
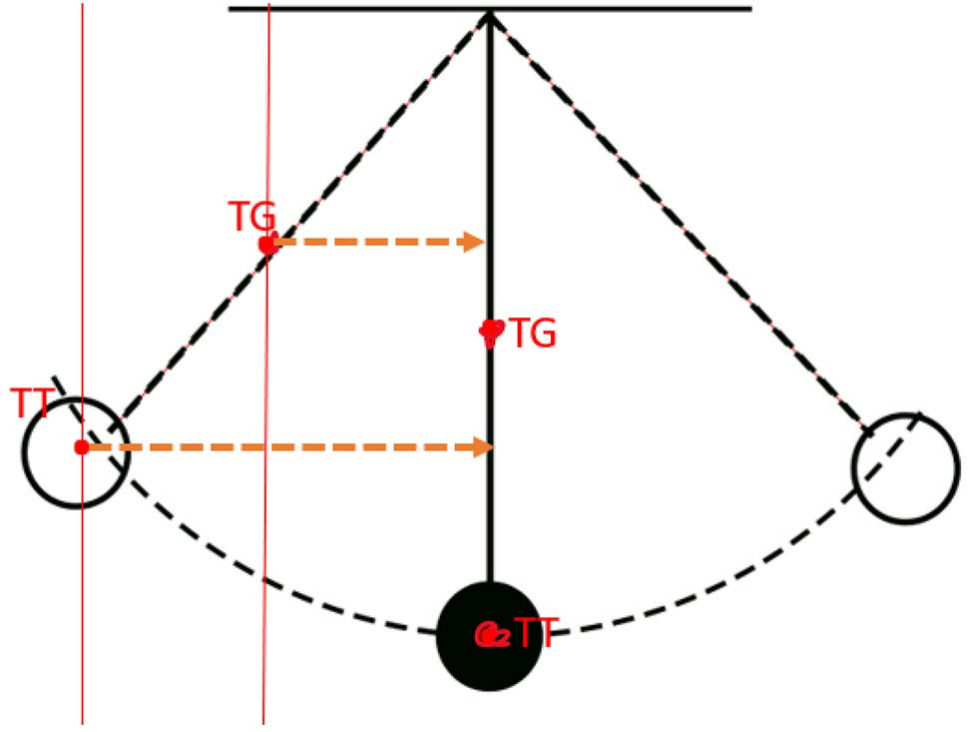
The pendulum effect of the osteotomy on TT-TG distance

Our measurements showed that for every 2° of varus opening up to 12°, the TT-TG distance would decrease by 1.7 mm. Geometrically, both anatomical landmarks (TT and TG) are distal to the rotation point. Thus, they remain in line no matter the amount of proximal correction. However, due to the so-called pendulum effect, the distance between the two points changes depending on the perspective. (Fig. 6)

Linear correlation of these two data points using the Pearson correlation coefficient was (r = -0,991). A very high negative correlation between varization and decrease in TT-TG distance (2-tailed, p < 0,001) was found.

## Discussion

The most important finding of this study was that the TT-TG distance decreased incrementally in patients with valgus knees undergoing 3D-CT simulated LDF-OWO, and for every 2° of varus opening up to 12°, the TT-TG distance would decrease by 1.7 mm due to the pendulum effect described in Fig. 6. This finding has implications for planning a concomitant tibial tuberosity transfer in patients with genu valgum, patellar instability and an abnormal TT-TG distance.

Correction of valgus alignment of the distal femur has been recognized as an important factor in treating patellar instability, and both lateral open and medial closed wedge osteotomies have been described. A recent meta-analysis has shown favorable clinical outcomes and low recurrent instability rates using both types of osteotomies [10].

Lateral opening wedge techniques may increase patello-femoral contact pressure, necessitate bone grafting, and have a risk of hinge fracture, collapse, and loss of correction. Lateral opening wedge osteotomies decrease patellar height and may help correct patella alta and allow for an easier trochleoplasty [10]. In contrast, closing wedge osteotomies have a reduced risk of nonunion and delayed union, a speedier recovery time, and no increase in patellofemoral contact pressure, but may interfere with MPFL reconstruction tunnels [11]. Correction of torsional deformities can also be performed during DFO.

Increased TT–TG distance is a well-known risk factor for acute patellar dislocations and has also been implicated in the failure of soft tissue reconstructions for patellar instability [12]. Changes in TT-TG distance following osteotomies around the knee have not been elucidated in detail. Sim et al. showed that the TT-TG distance was reduced following a medial open wedge high tibial osteotomy [13]. A case series of 23 knees analyzed the change in TT-TG distance following a medial closing wedge osteotomy for valgus correction in patients with recurrent patellar dislocation [6]. The authors found that the TT-TG distance significantly decreased from 20.4 to 13.5 mm after distal femoral osteotomy. Deng et al. analyzed 13 patients undergoing biplanar medial closed wedge DFO combined with MPFL reconstruction. They found that a significant decrease in TT-TG distance from 19.6 to 13.3 mm occurred without any intervention on the tibial tubercule [4]. In contrast, no significant change in TT-TG distance could be observed in 25 patients undergoing closing wedge distal femoral osteotomy and MPFL reconstruction [5].

Flury et al. performed a comparative (pre vs. post-operative) 3D computer mapping study of 10 knees undergoing biplanar medial closed wedge osteotomy and found that Q angle decreased after surgery and was correlated with the degree of varization [14]. However, the authors observed that change in TT-TG distance after varization was minimal and irregular and was probably due to unintentional rotation during surgery [14]. In contrast, our study was a virtual simulation of varization where unintended rotation was strictly avoided using the computer software. Varization was performed from a standard pivot point, not allowing for displacement in other planes. This ideal scenario of perfect varization without rotation produced using 3D computer modeling might not be reproducible in real life, given the complexity of boney malalignment in patients with patellar instability [12]. Therefore, past and future studies might report inconsistent changes of TT-TG distance after DFO.

Guided growth techniques with temporary or permanent hemiepiphysiodesis have been described in skeletally immature patients to correct valgus and patellar instability. Most of these studies do not utilize soft tissue procedures on the medial soft tissues and rely on gradual valgus correction to treat patellar instability [15]. Ceroni et al. proposed a trigonometric formula for predicting the correction of TT-TG distance after guided growth correction of valgus using hemiepiphysiodesis. They indicated that a 1 mm change in TT-TG distance would be expected for every varus or valgus correction degree [16].

We are not aware of a study analyzing the changes in TT-TG distance after a lateral opening wedge distal femoral osteotomy. Swarup et al. reported on eight patients undergoing lateral open wedge DFO combined with lateral retinacular release for patellar instability. Good clinical outcomes and correction of coronal alignment were reported, but pre- and postoperative TT-TG distance measurements were not performed [17]. Wilson et al. reported on 11 skeletally mature patients undergoing lateral opening wedge DFO to correct valgus alignment and patellar instability. Improved Kujala scores and correction of the coronal mechanical axis were found, and a decrease in the Caton-Deschamps index was observed, implying decreased patella alta. The mean pre-operative TT-TG distance was 21.5 mm. However, no measurement was made post-operatively [18]. Our 3D computer simulation study found that for every 2 degrees of varus opening of the distal femur using the most commonly utilized lateral open wedge osteotomy technique, the TT-TG distance decreased by 1.7 mm. The surgeon should be aware of this change and decide whether an additional tibial tuberosity transfer is needed. Following the correction of valgus with LDF-OWO, the addition of a tibial tubercle transfer may not be required in patients with a TT-TG distance in the grey zone of 14 to 20 mm [19]. The amount of medialization may be decreased for patients with a TT-TG distance over 20 mm if an LDF-OWO is performed first.

### Limitations

This study has several limitations. Only six knees were available for analysis due to the low prevalence of valgus knees requiring correction in adults with patellar instability; therefore although the study was adequately powered for correlation analysis, it may be underpowered for other parameters. Furthermore, the study was performed in patients with underlying bone and soft tissue risk factors for patellar instability and not sawbones, reflecting the actual clinical situation. It should be kept in mind that this is a pilot study analyzing feasibility and will pave the way to adequately powered larger studies.

Only the situation after LDF-OWO was analyzed, and this may not reflect the changes occurring in TT-TG distance using guided growth techniques or medial closing wedge osteotomies. Changes in the ligaments and soft tissues around the knee could not be considered using a 3D-CT simulation. However, no effect of soft tissue tension is expected on TT-TG distance.

The varization was performed with strict avoidance of unintended rotation in this computer simulation study. This might not be the case during surgery where unintended rotation may occur during opening of the osteotomy site.

We used standing orthoroentgenograms to evaluate the mechanical axis and supine CT scans for simulation of osteotomies. A weight bearing CT scan of the whole extremity could have better replicated the actual clinical situation, however this imaging technique was not available. A single observer performed the measurements, therefore the reliability and reproducibility of the measurements were not verified.

## Conclusions

Lateral opening wedge distal femoral osteotomy causes a decrease in TT-TG distance.The surgeon should be aware of the magnitude of this change (1.7 mm decrease for every 2° increment of varization) and consider modifying the tibial tuberosity medialization osteotomy in patients with patellar instability if indicated.

### Electronic supplementary material

Below is the link to the electronic supplementary material.

Supplementary Material 1

## Data Availability

The datasets used and/or analyzed during the current study are available from the corresponding author on reasonable request.
