# Supplementary material for: TT-TG distance decreases after open wedge distal femoral varization osteotomy in patients with genu valgum & patellar instability. A pilot 3D computed tomography simulation study
Source: BMC Musculoskelet Disord. 2023 Sep 25;24:753. doi: 10.1186/s12891-023-06832-w (PMC10519055; doi:10.1186/s12891-023-06832-w)
Supplement: Supplementary file 1 — Supplementary Material 1 [file 12891_2023_6832_MOESM1_ESM.docx]

Video 1: Avoidance of unintended rotation or translation during angular motion of the distal osteotomy fragment using interactive rotation option of the 3-Matic software
